# Temporal context and latent state inference in the hippocampal splitter signal

**DOI:** 10.7554/eLife.82357

**Published:** 2023-01-09

**Authors:** Éléonore Duvelle, Roddy M Grieves, Matthijs AA van der Meer

**Affiliations:** 1 Department of Psychological and Brain Sciences, Dartmouth College Hanover United States; University of Oxford & Stanford University United Kingdom; University of Oxford United Kingdom

**Keywords:** place cells, structure learning, hippocampus, state spaces, memory, trajectory-dependent firing

## Abstract

The hippocampus is thought to enable the encoding and retrieval of ongoing experience, the organization of that experience into structured representations like contexts, maps, and schemas, and the use of these structures to plan for the future. A central goal is to understand what the core computations supporting these functions are, and how these computations are realized in the collective action of single neurons. A potential access point into this issue is provided by ‘splitter cells’, hippocampal neurons that fire differentially on the overlapping segment of trajectories that differ in their past and/or future. However, the literature on splitter cells has been fragmented and confusing, owing to differences in terminology, behavioral tasks, and analysis methods across studies. In this review, we synthesize consistent findings from this literature, establish a common set of terms, and translate between single-cell and ensemble perspectives. Most importantly, we examine the combined findings through the lens of two major theoretical ideas about hippocampal function: representation of temporal context and latent state inference. We find that unique signature properties of each of these models are necessary to account for the data, but neither theory, by itself, explains all of its features. Specifically, the temporal gradedness of the splitter signal is strong support for temporal context, but is hard to explain using state models, while its flexibility and task-dependence is naturally accounted for using state inference, but poses a challenge otherwise. These theories suggest a number of avenues for future work, and we believe their application to splitter cells is a timely and informative domain for testing and refining theoretical ideas about hippocampal function.

## Introduction

### Why splitter cells?

A central goal in neuroscience is to explain how cognitive and behavioral phenomena arise from the collective activity of populations of neurons and the specific circuit and cellular mechanisms that shape that activity, down to the single-cell level. The rodent hippocampus, and related brain regions, have been a productive area of research in this respect. The discovery of place cells, head direction cells and grid cells led to compelling theories about how these cells may support spatial memory (Epstein et al., 2017; Grieves and Jeffery, 2017; Moser et al., 2015). In turn, the discovery of population-wide phenomena such as theta phase precession and ‘replay’ in hippocampal neurons has also led to breakthrough theories concerning the rapid encoding and subsequent retrieval of episodic-like memories (Buzsáki, 1989; Foster and Knierim, 2012).

However, a big gap still remains between central concepts in the cognitive neuroscience of memory on the one hand, and what we know about the single cell and ensemble firing patterns of the neurons thought to underpin those processes on the other. Experience, as reflected in neural activity, is not simply encoded, stored and retrieved verbatim, but is organized into knowledge structures associated with the hippocampus, such as contexts, maps and schemas that permit generalization and inference (Behrens et al., 2018; Morris, 2006; O’Keefe and Nadel, 1978). The advent of large-scale neural recordings and accompanying analysis tools has made it possible to probe how such knowledge structures are encoded in the population activity of neurons (Ebitz and Hayden, 2021; McKenzie et al., 2014). For instance, the geometry (i.e. similarity structure) of population activity patterns that encode different experiences or task conditions is thought to reflect computational tradeoffs such as that between pattern separation and pattern completion, or between mixed and specialized selectivity, which in turn determine the subject’s understanding of the world (Kumaran et al., 2012; Rigotti et al., 2013; Russo et al., 2020). Thus, representational geometries can provide a bridge between the single cell, neural ensemble and cognitive process levels, promising a true multi-level account of how cognitive phenomena are realized neurally (Chung and Abbott, 2021; Kriegeskorte and Wei, 2021; Urai et al., 2022).

‘Splitter cells’ in the hippocampus, mostly studied in rodents, provide access to a rich, deep, yet coherent view of how experience and task structure shape neural activity (for previous reviews, see: Ainge et al., 2008; Dudchenko and Wood, 2014). Splitter cells fire differentially on the overlapping segment of trajectories that differ in where the animal came from, and/or where it is going. These cells are colloquially referred to as ‘splitters’ because they distinguish (i.e. split) overlapping spatial trajectories at their shared segment (Dudchenko and Wood, 2014; Frank et al., 2000; Hasselmo, 2005; Wood et al., 2000). Importantly, they do so based on information that is not present in sensory or motor patterns at the time of the splitting effect, but instead appears to reflect the recent past, upcoming future, and/or inferences about the state of the environment. Such internally generated representations are a hallmark of cognition across different domains (as formalized in e.g. predictive processing architectures for perception and motor control, Keller and Mrsic-Flogel, 2018), suggesting that splitter cells are not only an access point into internal processes which can elucidate the core computations carried out by the hippocampus, but into principles of cognition more generally.

Our overall goal in this review is to bring together the extensive experimental literature on splitter cells with current theoretical ideas about hippocampal function. To do so, we first establish a common set of terms and scope on the splitter phenomenon, including relating single cell and ensemble levels (following section). Next, we identify and synthesize consistent findings from the experimental literature on splitter cells (section *Experimental results on splitter cells*). Then, we introduce the key theoretical ideas of temporal context and state splitting (section *Computational models of splitter cells and their function*), and apply these theories to the data (section *Model predictions and experimental data*). We summarize and conclude with open questions in the last section (*Conclusions and remaining open questions*).

### Splitting at the single cell and ensemble level

#### Single cell splitting

**Figure 1.**
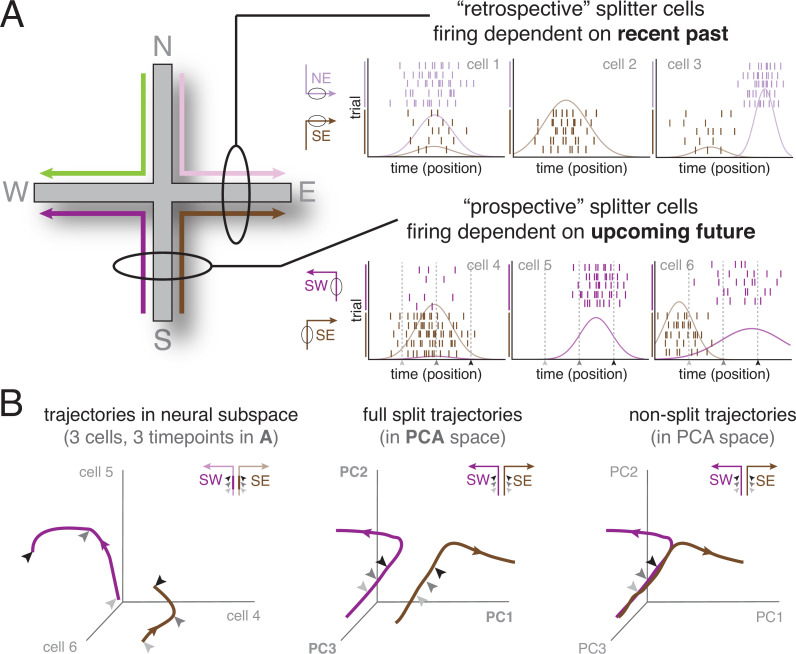
The splitter cell phenomenon at the single cell and ensemble levels. (**A**) Schematic activity of six idealized splitter cells during performance of a plus maze task (left), in which four different spatial trajectories are possible (SW, SE, NW, NE). The firing of a true place cell, encoding current location only, does not depend on past or upcoming trajectory; however, splitter cells distinguish recent past (e.g. NE vs SE, top row; depicted cells fire in E) and/or upcoming future (e.g. SW vs SE, bottom row; depicted cells fire in S). Note that individual splitter cells may fire in the same place for both trajectories, but show a difference in firing rate (left column), fire for only one of the trajectories (middle column), or fire in different locations (right column) depending on trajectory. Rasterplots show spikes (tickmarks) for a number of trials of each trajectory (color-coded) as well as tuning curves averaged across trials. For simplicity, we assume subjects travel at constant speed so that time and position are equivalent. (**B**) *Left*: Schematic neural activity trajectories of multiple splitter cells in ensemble activity space for the same trajectories as cells shown in the bottom row of (**A**); SW, purple; SE, brown. Each of the three axes corresponds to the activity (firing rate) of one cell. Arrows are matched with corresponding arrows in (**A**) and indicate three different time points (locations) during each trial. Note that the SW and SE trajectories occupy clearly distinct areas of neural activity space, even though the subject is traversing the same area of space (same three locations, indicated by arrows, along the S arm), ‘splitting’ trajectories based on different upcoming futures (E vs W). *Middle*: Schematic neural activity trajectories for a population of neurons, obtained by projecting onto the first three principal components of ensemble activity. Full trajectories are now shown, enabling comparison of ensemble activity during the common segment of the trajectory (S arm indicated by arrows; distinct but relatively close) and the diverging segment (E vs W arm, separating further in ensemble space). *Right*: Hypothetical ensemble activity in the *absence* of a splitter signal, for comparison. Note how neural activity trajectories on the common segment (the S arm) overlap, indicating SE and SW trajectories are not distinguishable until the E vs W arms are actually entered.

Splitter cells were originally reported in two independent studies using two closely related tasks in rats: alternation on a continuous ‘figure-of-eight’ T-maze (Wood et al., 2000) and alternation on a continuous W-maze (Frank et al., 2000). In both cases, the central stem of the maze is shared between two trajectories that differ in their recent past (coming from the left or the right) and/or in their upcoming future (going left or right). We illustrate the splitter phenomenon here using the plus maze from a subsequent study (Ferbinteanu and Shapiro, 2003), which enables an elegant comparison of neural activity on the shared segment of trajectories with different pasts (e.g. NE vs SE) or different futures (e.g. NW vs NE; Figure 1a). These and numerous other studies, which we will discuss below, revealed hippocampal cells that fired differentially depending on where the animal came from (**retrospective** splitters) as well as cells that fired differentially depending on where the animal was going next (**prospective** splitters).

For the purposes of this review, we group together the existing terms *trajectory coding* (Berke et al., 2009; Frank et al., 2000; Ito et al., 2015), *differential activity* (Ainge et al., 2007b; Grieves et al., 2016; Wood et al., 2000), *context-dependent activity* (Ainge et al., 2008; Ainge et al., 2007a; Dayawansa et al., 2006), *journey-dependence* (Bahar and Shapiro, 2012; Ferbinteanu et al., 2011; Ferbinteanu and Shapiro, 2003; Takahashi, 2013), and *splitter cells* (Dudchenko and Wood, 2014; Hasselmo, 2005; Kinsky et al., 2020; Levy et al., 2021; Zhao et al., 2022) as instances of the same phenomenon, which we name here by the colloquial term ‘splitters’. This term references the computational process of state-splitting (discussed in more detail in section *Computational models of splitter cells and their function*) which is at the center of leading theoretical accounts of hippocampal function, while sidestepping the more subtle distinctions between for example trajectory and journey (see Box 2). In the majority of studies, splitter cells are defined as the trajectory-dependent subset of place cells on the overlapping part of the maze, such as the central stem of a continuous T-maze (see Box 3 for variability in definitions). However, **we consider a splitter any cell that is active during, and distinguishes between, the overlapping part of different trajectories,** including cells active at a specific time during a delay (‘time cells’ or ‘episode fields’, Gill et al., 2011; MacDonald et al., 2011; Pastalkova et al., 2008).

Note that the splitter cell phenomenon concerns ‘in-field’ activity, that is, firing rates thought to reflect current, *ongoing experience*. This contrasts with ‘out-of-field’ activity, such as occurs during sharp wave-ripples (Buzsáki, 2015; Wilson and McNaughton, 1994; for review, see Pfeiffer, 2020), and also with theta sequences (Foster and Wilson, 2007; Johnson and Redish, 2007; Wang et al., 2020a). These phenomena can also be regarded as prospective or retrospective, such as when replaying a place field sequence associated with an upcoming left or right trajectory; however, unlike splitter cells, they require the participating cells to have a firing field on a non-overlapping segment. Thus, theta sequences and replay, although both can and likely do involve the activity of splitter cells (Takahashi, 2015; Tang et al., 2021), are distinct phenomena that we do not discuss further here.

To date, dozens of studies have reported the splitter phenomenon. Typical percentages vary between 10% and 60% of place cells active on the overlapping segment (usually the central stem) showing the splitting effect; however, there is significant variability across, and in some cases within, studies (discussed in section *Variability of the splitter signal across tasks and studies*). The effect is found on a variety of tasks, including different variants of continuous T-, Y- and radial arm maze tasks, and tasks with discontinuous trajectories such as plus mazes and double-Y mazes (Ainge et al., 2007b; Grieves et al., 2016). Splitting can also be seen during delays that occur on the overlapping segment of distinct trajectories (e.g. Ainge et al., 2007a; Hallock and Griffin, 2013; Pastalkova et al., 2008), and even in the absence of an explicit task (Keinath et al., 2020). Although we focus here on spatial trajectories, splitting has also been observed on non-spatial tasks such as those presenting overlapping sequences of discrete odor cues (Allen et al., 2016; Ginther et al., 2011; Shahbaba et al., 2022) indicating that the phenomenon is quite general. Place cells have also been found to have differential activity on the two travel directions of a linear track (‘directional place cells’, McNaughton et al., 1983; Battaglia et al., 2004); as we discuss below, this phenomenon may result from similar underlying processes as splitter cells, but we do not explicitly consider it here since sensory information and head direction are different for the two directions. Similarly, although typical splitter studies use tetrode recordings in rats, trajectory splitting has also been found using calcium imaging in mice (Kinsky et al., 2020; Levy et al., 2021; see also Nieh et al., 2021; Keinath et al., 2020; Sun et al., 2020), using recordings in macaques (Bretas et al., 2019; Gulli et al., 2020) and with intracranial recordings (Ekstrom et al., 2003) and fMRI in humans (Brown et al., 2016; Brown et al., 2010; Chanales et al., 2017; see also: Hsieh et al., 2014).

#### Ensemble splitting

Takahashi, 2013 was the first to implement the idea that at the population level, splitter cell activity can be conceptualized as a different **pattern** of activity at the same spatial location, with ensemble similarity ‘pulled apart’ (i.e. split) by a hidden variable. Each location along a spatial trajectory experienced by the animal is associated with a specific set of place cell firing rates, which occupies a point in neural activity space where each axis indicates the activity of one neuron (Figure 1b, left). As the animal moves in space, neural activity changes to form a trajectory in neural space. Nearby points indicate similar population activity, and far away points indicate different activity. For more than three neurons, dimensionality reduction techniques such as principal component analysis (PCA) can be used to visualize activity and ensemble similarity of the entire recorded population of neurons. Splitter activity manifests as a separation in neural activity space, even though the animal is physically in the same location (different activity in the South arm for SE and SW trajectories; Figure 1b, middle). For comparison, if there was no splitter effect, the neural activity in the South arm would be indistinguishable (i.e. not split) in neural activity space (Figure 1b, right).

This ensemble view has not historically been applied to splitter cell activity, but it is a helpful addition for a number of reasons. First, it offers a visually intuitive view of the phenomenon and indicates its strength (the extent of splitting) by distances in neural activity space, which also makes it a useful basis for quantification in data analysis. Second, it provides additional statistical power: a single cell might not pass a significance test for say, splitting SE vs SW, but if many cells show that same tendency, there may be a robust population-level difference (e.g. Keinath et al., 2020). Third, the ensemble similarity view enables easier comparisons with other types of data, such as that collected with MEG or fMRI, which cannot access single neuron activity but do provide population-level measures. Finally, physiologically speaking, downstream neurons and brain structures receiving inputs from these hippocampal neurons ‘see’ a population activity pattern, thus it provides a more complete picture of the ways the signal can be interpreted. Accordingly, ‘splitter cell’ is not an anatomically, genetically or physiologically hardwired cell type in the way that e.g. a specific type of interneuron is (however, it appears they may be anatomically segregated, likely due to different anatomical inputs – see Harvey et al., 2022). Single-cell splitter activity is not a fixed property, but rather a reflection of, and a contributor to, an ensemble phenomenon. This notion is supported by studies that compare the activity of splitter cells across different tasks or conditions and find that a cell can be a splitter in one condition but not the other, or change its coding category (prospective vs retrospective) across tasks (e.g. Ferbinteanu et al., 2011; Bahar and Shapiro, 2012). Thus, in this review we use the term ‘splitter signal’ to refer to population-level activity that distinguishes between different spatial trajectories at a point of overlap.

What, if anything, distinguishes the ‘splitter’ signal from time cells (MacDonald et al., 2011)? In general, the activity of all these cells reflects a combination of sensory and internally driven components. A major motivation for examining splitters is that it is an access point into internally generated activity during encoding, because all sensory cues at the time are identical. Time cells are similar to splitter cells in the sense that they show a large internally generated component, especially when sensory input is ‘clamped’ by running on a treadmill (Kraus et al., 2013; MacDonald et al., 2011; Pastalkova et al., 2008; Yong et al., 2022). Note that "Flickering" between multiple maps or reference frames (Jackson and Redish, 2007; Jezek et al., 2011; Kelemen and Fenton, 2010) and theta sequences (Foster and Wilson, 2007; Skaggs et al., 1996) similarly are not sensory-driven. However, the encoding of time, by itself, does not distinguish between different past and/or future experiences; rather, like place cells, time cells offer a scaffold on which such experiences can be differentially encoded. Place cells and time cells can both be splitter cells, as long as they meet the criterion of showing **trajectory-dependent activity** at a tightly controlled point of overlap such as the central stem of a maze or a treadmill. Thus, we consider both in this review, with particular focus on the dorsal CA1 area of the rodent hippocampus, where most splitter studies have been conducted, but we provide a broader anatomical view in Box 1.

Box 1. **Splitter cells beyond the hippocampus.**

This review focuses on dorsal CA1 in rodents, where most splitter cell studies have been performed, but place cells that split trajectories are also found in dorsal CA3 (Allison, 2016 - PhD thesis; Bahar and Shapiro, 2012; Ito et al., 2015; Keinath et al., 2020; Senzai and Buzsáki, 2017), dentate gyrus (Senzai and Buzsáki, 2017), subiculum (Kitanishi et al., 2021) and medial entorhinal cortex (Frank et al., 2000; Gupta et al., 2012 (in low proportions); Lipton et al., 2007; O’Neill et al., 2017). Single neurons in areas outside the hippocampal formation generally do not show sharply tuned location-specific firing, but a number of anatomically related regions show trajectory-dependent activity, including medial prefrontal cortex (mPFC; Baeg et al., 2003; Euston and McNaughton, 2006; Fujisawa et al., 2008; Ito et al., 2018; Ito et al., 2015; Jones and Wilson, 2005; Jung et al., 1998; Shin et al., 2019; Stout and Griffin, 2020; Tang et al., 2021; Yang and Mailman, 2018), specifically, the anterior cingulate cortex (ACC; Cowen et al., 2012) orbitofrontal cortex (Young and Shapiro, 2011) nucleus reuniens (NRe; Ito et al., 2018; Ito et al., 2015), posterior parietal cortex (Harvey et al., 2022), striatum (Mizumori et al., 2004; Regier et al., 2015) and retrosplenial cortex (Chinzorig et al., 2020; Miller et al., 2019; Vedder et al., 2017). Finally, the firing of head-direction cells (from the anterodorsal and laterodorsal thalamic nuclei) can also be trajectory-dependent (Enkhjargal et al., 2014). Note that the anatomy of the prefrontal cortex / hippocampus circuit is quite complex and involves the anterior thalamus as well as the nucleus reuniens (see Porter and Bilkey, 2015, blog post).

The dependencies between trajectory-splitting in these different regions have not been fully characterized, but a few studies examined the effects of lesions/inactivations in selected regions on CA1 splitting. Inactivations of CA3 (Keinath et al., 2020), mPFC (Guise and Shapiro, 2017), and nucleus reuniens (Ito et al., 2015) all reduce the CA1 splitter signal, while lesions of the medial entorhinal cortex do not impact splitter coding in CA1 (Sabariego et al., 2019). Silencing the supramammillary nucleus reduces splitter coding in nucleus reuniens and dCA1, but not in mPFC, suggesting that it stops the transmission of the mPFC contribution to CA1 splitting (Ito et al., 2018). Thus, it seems there are multiple contributions to the splitter signal, of which the mPFC-NR-CA1 pathway is the best characterized, but not the only factor.

The relatively specific effects of NRe lesions on prospective, but not retrospective, splitters (Ito et al., 2015) is particularly interesting in the light of their different theoretical underpinnings (see sections *Computational models of splitter cells and their function* and *Model predictions and experimental data*). Conversely, inactivations of CA3 disrupt retrospective firing (Keinath et al., 2020) but it is unknown if they disrupt prospective firing. It remains to be tested if disruptions of other nodes in this circuit similarly have different effects on these two cell types. The contributions of the lateral entorhinal cortex, which contains signals that look very much like the decaying memory traces required by the temporal context model (Tsao et al., 2018; see section *Computational models of splitter cells and their function*) also are yet to be investigated.

## Experimental results on splitter cells

### What do splitter cells split?

While differences in past or future *trajectory* are perhaps the most obvious feature that could be underlying the splitter signal, it is by no means the only one (see Box 2). Current *goal*, that is the spatially defined location that the subject is navigating towards, is another. A different possibility from spatially defined features such as trajectory and goal is the encoding of *task states*, one state for each possible task configuration that disambiguates its sensory and response characteristics. In T-maze alternation, the task can be in two possible states, left-rewarded and right-rewarded, but the state labels are arbitrary, and actually maintain no spatial representation of left and right. All that task state representations need to do in this case is to distinguish between configurations that determine whether or not reward will be delivered at a given reward site. In other words, state representations need not have any particular representational structure; in contrast, spatial representations *do* have requirements on their representational structure. In order to infer whether location A is close to location B, arbitrary labels are not sufficient, because there is no operator that converts two arbitrary labels into a spatial distance (unlike, say, for a 1-D coordinate system, where a simple subtraction yields distance). State representations have minimal requirements on representation structure, in that all they need to do is identify A as being different from B. We discuss this idea further in section *Computational models of splitter cells and their function*. Finally, the splitter signal may encode the current *policy*, that is the mapping from situation (combination of currently available sensory cues and task state) to action, like ‘turn left at the choice point’.

Box 2. **Key terms and concepts in thinking about splitter cells.**

Goal: in spatial navigation tasks, refers to the currently rewarded location. If the task is well-learned, this usually, but not necessarily, corresponds to the subject’s intended destination. This term can be confusing because the existence of a goal defined by the task — for instance, ‘the feeder on the left arm is currently baited’ — need not imply that the subject has a representation of that goal, even when it successfully gets there. This is so because its behavior may be driven by a stimulus-response strategy (e.g. ‘turn left at the choice point’) that has no knowledge of the resulting outcome. For more information, see the extensive literature on goal-directed vs. habitual behavior (Balleine and Dickinson, 1998) and place vs. response strategies (Packard and McGaugh, 1996; for reviews, see Arleo and Rondi-Reig, 2007; Goodman, 2021; Nyberg et al., 2022).

Trajectory: position as a function of time, in other words, a path through space. Note that the subject need not represent its entire trajectory, i.e. extending fully back to its point of origin and fully towards the upcoming goal; it can be more temporally restricted, and may be oriented exclusively in the past or future. Some studies make a distinction between trajectory and *journey*, such that a journey is defined only by the start and end points of a trajectory regardless of the specific trajectory taken in between. For instance, starting from the N arm of a plus maze, going into the S arm, then turning around before going to the end of the E arm would be a NSE *trajectory* but a NE *journey* (Ferbinteanu and Shapiro, 2003).

Task state: discrete task configuration that fully specifies the current sensory properties of the environment (e.g. any cues that may be on) as well as how the task will respond to the subject’s actions, such as whether a given location will be rewarded or not, or what (if any) specific trajectory needs to be taken to yield reward. Task states can be *overt* (e.g. if tone sounds, then a lever can be pressed for reward) or *latent* (not directly perceivable, e.g. ‘left rewarded’ and ‘right rewarded’ states in T-maze alternation). Note that task states are not given to the subject, but must be learned from experience, and the state representation that the subject learns can diverge from the true task states (see section *Latent state inference*).

Policy: defines the actions taken by the subject in each state, e.g. ‘turn left at the choice point’. Note that state here means the subject’s own internal state representation, which may or may not align with the true task state. Policies may be encoded as a sequence of egocentric turns, or as a mapping between situations and actions.

Examples:

- The same goal (end of West arm) can be reached by different trajectories (NW, SW).
- Task state may be perfectly aligned with goal (‘W is rewarded’) but need not be, e.g. other possible task states include how many laps need to be run before reward is delivered, or whether the active task rule is currently win-stay or win-switch.
- A policy like ‘turn left at the choice point’ will result in different trajectories depending on the start point (N, S; response strategy). This simple policy could be augmented with a more specific state representation, for instance ‘turn left when at the choice point and facing north, turn right at the choice point when facing south’ (place strategy).
- In T-maze alternation, the environment has two experimenter-defined task states (left rewarded; right rewarded). But the subject could learn instead, erroneously, that there is only one task state, with each side having a 50% probability of being rewarded when choosing randomly.

**Box 2—figure 1.**
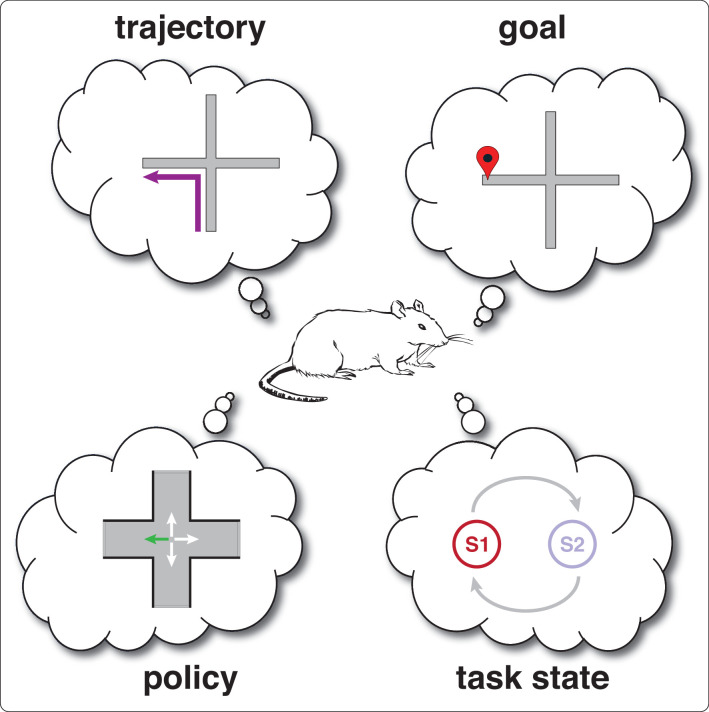
Schematic illustration of different concepts (features) relevant to splitter cells.

Note that the canonical tasks used for splitter cell studies (W-, T- or plus mazes) may distinguish some, but typically not all, of these possibilities. For instance, the existence of prospective splitters on the plus maze (SW vs SE; Figure 1) could be the result of encoding a difference in goal (W vs E), policy (turn left or right), task state (is W or E rewarded) or trajectory (SW vs SE). Retrospective splitters (NE vs SE; Figure 1) can’t be explained by goal or task state (E is rewarded in both cases) but still could be either past trajectory (N vs S) or policy (turn left for NE, right for SE). In general, dissociating these possibilities requires specifically designed tasks, and it is difficult to dissociate them all in a single study (but see the maze suggested in Figure 3).

In tasks where the *trajectory* to the goal is relevant, for example two trajectories to the same goal with only one trajectory rewarded at a given time, splitter cells encode different trajectories that ended in the same goal (Grieves et al., 2016; Ito et al., 2015). These experiments show that there is a clear component of prospective splitter activity that cannot be attributed to differences in goal location; in other words, differences in goal location are not a necessary requirement for splitters. Note however that Bower et al., 2005 found no splitters in one of the three tasks they studied, which had different trajectories to a common goal. We discuss some possible reasons for this variability across studies in the next section *(Variability of the splitter signal across tasks and studies)*. In addition, experiments with multiple choice points, such as the double-Y maze, have shown that splitter activity does not only depend on the upcoming choice (left or right) but discriminates the full trajectory, for example left-left vs left-right in Ainge et al., 2007b.

However, apart from demonstrating that differences in goal are not sufficient to generate the splitter signal, the relative contributions of other factors, such as trajectory, task state, and policy, have not been clearly determined. Disentangling their contributions is challenging because of how interrelated they are: changing the goal location changes at least some part of the trajectory, and differences in trajectory are typically correlated with the specific sequence of actions (i.e. egocentric turns) that the animal takes, so the notion of ‘policy’ (i.e. what action to take in what situation) would change together with differences in trajectory. Most perniciously, any of these are also changes in task state. How can such closely related notions be dissociated? There may still be some mileage in the analysis of existing data, for instance in asking whether there are cells on the plus mazes or double-Y mazes that fire similarly for the same past or future action (e.g. turn left at the choice point) regardless of current location; this would be indicative of policy coding. More generally, a promising approach afforded by taking an ensemble view of the splitter signal is to use representational similarity analysis (RSA) (Kriegeskorte et al., 2008; McKenzie et al., 2014). The intuition underlying this approach is that states are discrete and only need to serve as labels without any similarity structure; therefore, each state representation is expected to be equally different neurally from any other state. In contrast, *goals* and *trajectories* are spatially represented, so if there is more overlap between 2 trajectories or if 2 goals are closer, then they are also expected to be more similar neurally. This is only up to a point, since there is evidence that memory traces/trajectories that are very similar are actively decorrelated in the hippocampus, presumably to prevent interference, e.g. Favila et al., 2016; for review, see Yassa and Stark, 2011. We develop this idea further in section *Model predictions and experimental data*.

### Variability of the splitter signal across tasks and studies

From the earliest reports, a notable and confusing feature of the splitter cell literature has been that there are **large differences in strength of the splitter signal across studies**, as measured by the percentage of trajectory-coding cells out of active place cells (e.g. 16% retrospective and 3% prospective in Frank et al., 2000 compared to 67% overall in Wood et al., 2000; note that these percentages correspond to analyses attempting to control for behavioral confounds in both studies). Do these differences tell us something meaningful about the properties of these cells and cognitive processes in the hippocampus? Or, alternatively, are they due to experimental differences such as recording location and behavioral task, and/or methodological issues such as differences in how possible confounds are handled and what statistical criteria are applied?

In general, the splitter cell phenomenon is *sensitive to differences in methodology and analysis criteria* across studies. For instance, one obvious possible source of a putative splitter signal is if the animal has subtly different positions and head directions on the central stem when it is about to go left, as compared to when it is about to turn right. Some studies attempt to control for this, while others do not. Box 3 discusses some of the common methodological and analysis differences that can make it challenging to compare across studies. As a result of this overall issue, the most informative studies tend to be those that compare multiple experimental conditions within the same study, so that methodological factors are unlikely to be the reason for any differences observed. We review key results from these and other across-task comparisons next.

Box 3. **Detection methods and ruling out sensory confounds in detecting splitter cells.**

We present a few caveats relevant when comparing splitter signal strength across studies:

1. Behavioral and sensory confounds. Hippocampal neurons are known to be affected by multiple parameters: location, but also running speed (McNaughton et al., 1983), running direction (Jercog et al., 2019; Muller et al., 1994), and many other sensory parameters including, but not limited to, barriers (Muller and Kubie, 1987), floor texture (Wang et al., 2020b), and contextual information such as the color (Jeffery and Anderson, 2003) or the odor of an environment (Anderson and Jeffery, 2003). True splitter coding should exist independently of these factors, which is why it is important to control for any potential sensory difference between the two conditions that are 'split' over (usually different trajectories), as well as location, movement direction and speed.

When using linearized positions or bins (as is typically done in splitter studies), subtle differences in location, head direction and speed may be overlooked, causing firing differences between overlapping trajectories to be falsely interpreted as splitter firing. These effects can be substantial: e.g. Wood et al., 2000 found that 94% of place cells active on the central stem of their continuous T-maze had significantly different firing between left and right trajectories, but when controlling for running speed, head direction and lateral position, this proportion dropped to 67%. In the most extreme attempt to control for behavioral nuisance variables, Dayawansa et al., 2006 had a head-fixed rat running on a treadmill that was itself on a motion stage, such that the entire treadmill could be moved around in a maze, thus perfectly controlling the position, head-direction, and speed of the animal. They nevertheless found a very large proportion of splitter cells (82%); however, their splitter detection method was different from that of most studies (categorizing cells which did not show a significant correlation between activity in the 2 routes as splitter cells, instead of cells with a significant difference in firing rate between the two routes), making comparisons difficult. Splitter percentages from studies that do not control for these behavioral parameters should be interpreted conservatively (e.g. Levy et al., 2021; Shin et al., 2019; Tang et al., 2021).

2. Remapping. A change in context, defined as a 'complex set of environmental cues that influence learning and behavior' (Kubie et al., 2020) can cause partial or global remapping as mentioned above (for recent reviews, see: Kubie et al., 2020; Sanders et al., 2020). Time passed, or the animal being taken out then back in the environment, can change place cells' firing (in rats: Duvelle et al., 2021; Mankin et al., 2015; in mice: Keinath et al., 2020; Ziv et al., 2013). These effects may interfere with splitter detection, for example when attempting to detect consistent splitter firing across multiple sessions (Hallock and Griffin, 2013) or comparing different strategies which are run in blocks (Eschenko and Mizumori, 2007). An A-A-B-B-A or similar design is helpful in ruling out the effects of time.

3. Detection methods used in different studies might also be different from one study to the next, complicating comparisons of splitter counts or percentages. The most commonly used method is an ANOVA or ANCOVA that looks for significant differences in firing activity between the conditions of interest (Wood et al., 2000). The use of GLMs is particularly appropriate because it enables the modeling of nuisance variables/possible confounds, as well as the distribution of single cell firing rates. Permutation analyses are also used, with a criterion applied to one or more spatial bins of the central stem, which can be continuous or not. Some studies look for rate remapping while others may look for spatial remapping (change of place field location), or both. Studies may use different significance thresholds for their analyses, e.g. 0.05 in Wood et al., 2000 vs. 0.01 in Frank et al., 2000 for some analyses. Some studies varying the task within a recording may only consider splitter cells those reliably discriminating trajectories across all tasks instead of only within a task Hallock and Griffin, 2013; most studies will only categorize as splitters those neurons that have already been categorized as place cells, while others will only look for trajectory coding without selecting for spatial coding first (e.g. Kitanishi et al., 2021; Levy et al., 2021).

#### Differences in task demands and/or strategy

An intuitive idea is that the splitter signal might be reflective of a **memory demand**: in alternation tasks, a memory of the previous trial is required to determine the currently correct choice, whereas on a cued version of the same task, no memory of the previous trial is required. Thus, we may not expect splitter effects on cued tasks, which have no memory requirement other than the association between the cue and the reward. To test this idea, Ferbinteanu et al., 2011 trained the same rats on both a cued and a memory-guided (place) task on the same plus maze. Thus, the trajectories across these tasks were behaviorally identical but had different memory demands. Nevertheless, proportions of retrospective splitters were the same for both tasks (40%) as were those for prospective splitters (20%). Interestingly, changing the task affected prospective cells more than retrospective cells, with about half of the prospective cells (start arm splitters) in one task ceasing to be a splitter in the other task, while more than 80% of the retrospective splitters kept their category in both tasks.

Similarly, a number of studies have specifically varied memory demand, by comparing the same memory tasks with different delays, rather than comparing different tasks (e.g. place vs cue) which may also change the strategy being used by the animal. -These often find comparable splitter strength between continuous and delayed alternation versions of the same T-maze task (e.g. Takahashi, 2013 found no difference between continuous alternation, delayed alternation and also cued navigation), or between working memory and reference memory tasks on a radial arm maze (Xu et al., 2019). Hallock and Griffin, 2013 also compared continuous, delayed and cued T-maze tasks, but they did not compare splitter strength across them, focusing instead on identifying cells that were “consistent splitters” across tasks, which they did not find evidence for (consistent with an ensemble interpretation rather than hardwired splitters). In contrast, Robitsek et al., 2013 found the differential signal to be stronger in a (30 s) delayed alternation compared to a continuous alternation (in which the rats were trained first).

Thus, in studies that compare splitters across tasks with different memory demands or strategies in the same animals, it seems the splitter signal is generally comparable, with no clear effect of memory demand. However, the picture becomes more complicated when comparisons across tasks run with different animals are made. In Griffin et al., 2012, data was reported from rats trained on a cued (visuo-tactile discrimination) task only, as compared to 1 other rat trained on a continuous alternation task only. Little prospective splitter coding was apparent in rats who did the cued task (17%) compared to the rat on the alternation task (58%). In support of the idea that less memory-demanding tasks learned in isolation may recruit less splitter cells, Berke et al., 2009 reported essentially no splitter activity — whether prospective or retrospective — on a continuous plus maze where the rewarded arm was indicated by a light cue. A notable procedural difference in Berke et al., 2009, compared to Ferbinteanu et al., 2011, is that in the former, trials starting and ending at different locations on the plus maze were interleaved, whereas in the latter, they were run in blocks. As we discuss in the *Latent state inference* section, this difference could influence how animals structure their internal representation of the task and the environment.

In a seemingly surprising twist, Ainge et al., 2007a compared continuous and delayed alternation on the same maze, again in different groups of rats. Even though the delayed task had higher memory demand, essentially no splitter cells were found on the central stem (4–15% depending on the analysis) while on the continuous task, splitters were abundant (41–44%). This may seem paradoxical, but may make more sense when considering that they did find splitters during the delay (32%).We will discuss an interpretation of this set of findings from a modeling perspective in the section *Model predictions and experimental data*. Confusingly, Robitsek et al., 2013 did find splitters on the central stem after a delay (58%), but unlike Ainge et al., 2007a these animals were trained on both a continuous and a delayed version of the task, which may explain the difference.

In conclusion, although there have not been many studies that make direct comparisons, it does seem that when several tasks are learned by the same rats, similar proportions of splitter cells are found in all of them regardless of memory demand. There is some suggestion that when only one task is learned, trajectory splitting only appears in those tasks requiring spatial or working memory, but this issue would benefit from more systematic examination with larger numbers of animals and with procedural differences controlled for.

##### Differences in hippocampal involvement

In addition to memory demand, a related possibility that may underlie the variability in the splitter signal across tasks and studies is whether or not that task requires the hippocampus. Is there any correspondence between which tasks depend on the hippocampus, and the strength of the splitter signal? Perhaps tasks that are not hippocampus-dependent do not show hippocampal splitting. One caveat with this approach is that redundancies between multiple systems can make interpretation difficult: as discussed earlier in this section, multiple studies find splitters on tasks that are likely hippocampus-independent, like the cued task in Ferbinteanu et al., 2011. In that case, while it’s interesting that the hippocampus still shows splitters, it doesn’t speak to the issue of whether that splitter activity is supporting behavior, because the hippocampus may or may not be used for behavior. However, if a task requires splitting (i.e. has an overlapping segment that needs to be disambiguated in order to solve the task) and requires the hippocampus, yet no splitter cells are found, that would argue against the notion that its splitters specifically are important for the behavior — this is the informative case.

As mentioned above, Ainge et al., 2007a find exactly this. They first replicated Wood et al., 2000 finding that on a continuous T-maze without a delay, splitter cells on the central stem were numerous. They then showed that performance of this continuous version of the task does *not* depend on the hippocampus, but that with the addition of a delay at the base of the central stem, performance *does* become hippocampus-dependent. Surprisingly, in this hippocampus-dependent version of the task, splitting activity on the central stem was all but abolished (without a change in the number of place fields there), although significant trajectory splitting was found during the delay period. Thus, this study confirms that a task need not depend on the hippocampus for splitter cell activity to exist, and further demonstrates that hippocampal splitter cell activity *on the central stem* is not required for successful task performance, even if the task itself is dependent on the hippocampus.

This result is surprising, because on the one hand, it seems hard to argue that hippocampal activity is supporting the decision when activity coming up to the choice point doesn’t differentiate upcoming decisions; yet, on the other hand, the task is hippocampus-dependent. How to reconcile these two observations? One hypothesis is that the hippocampus is required to carry activity across the delay, but then sets up a policy elsewhere (e.g. ventral striatum, medial prefrontal cortex) once the run up the central stem starts. This account would predict that on the delay version of the task, disruption of hippocampus on the central stem (e.g. with optogenetic inhibition, as was done for NRe in Maisson et al., 2018) would have no effect, but stimulation during delay should have an effect. Conversely, disruption of e.g. striatum would have no effect early during the delay, but would have an effect when applied on the central stem run before the choice point. A different possibility is that dorsal hippocampus doesn’t have a sufficiently long integration timescale to show splitters on the central stem following the delay, but a subregion with longer integration time, putatively ventral hippocampus, would show splitters there (we develop this multiple-timescale idea, which remains to be tested, in more detail in the section *Temporal context models* below).

##### Differences in the amount and type of experience with the task

A further possible factor underlying differences in the strength of the splitting signal between and within studies is the amount and type of training that the animals received on the task. A commonly observed effect across studies is that the strength of the splitter signal increases gradually with experience (third task in Bower et al., 2005; Gill et al., 2011; Huang, 2010 - PhD thesis; Kinsky et al., 2020; Levy et al., 2021 - imaged across 18 days; Stevenson, 2011 - PhD thesis). Few studies have examined differences in the time course of prospective and retrospective splitting, although Shin et al., 2019 compared early and late sessions, run on the same day on a W-maze task, and found the increase in percentage of retrospective cells was larger than the increase in prospective splitters.

In contrast to this gradual emergence of splitting, in Lee et al., 2006 the proportion of splitters did not increase over 4 days/sessions. However, in this study, rats were pre-trained to run unidirectional laps (using a barrier blocking the other arm) and recording day 1 was the first day they were allowed to visit the opposite arm and alternate. This study highlights an early idea about how experience may affect the splitter signal: whether and how barriers were used during training. In several tasks finding *low* percentages of splitter cells in dorsal CA1, barriers were not used during learning, such as in the first (‘complex-sequence’) task in Bower et al., 2005, and others (Frank et al., 2000; Griffin et al., 2012; Hallock and Griffin, 2013 - barriers used for one training day; Lenck-Santini et al., 2001- no splitters; Regier et al., 2015). Conversely, barriers were used during learning of tasks which then showed *high* splitter counts (>30%), such as Wood et al., 2000, Lee et al., 2006, the continuous alternation task of Griffin et al., 2012; Takahashi, 2013, and the ‘barrier-trained’ task in Bower et al., 2005. This difference makes sense in the light of “latent state” accounts of hippocampal activity, as we discuss in the section *Model predictions and experimental data*. An alternative explanation is that barriers placed in an asymmetric way for each trial type would trigger local place cell remapping, which would then leave a trace in the place cell signal even after barrier removal, thus creating two different activity patterns for the two states (e.g. Deshmukh and Knierim, 2013; Vandrey et al., 2021). A further difference between studies using barriers is whether rats are pre-trained with barriers but never perform barrier-forced alternation trials,as in e.g. Griffin et al., 2012, or if they learn to alternate with barriers, as in e.g. Wood et al., 2000. However, we also note that barriers are not the only relevant factor: some studies find high splitter cell counts even though no barriers were used (e.g. Ainge et al., 2007b; Catanese et al., 2014; Catanese et al., 2012; Grieves et al., 2016; Ito et al., 2015).

### Relationship with decision-making

The relationship between splitter cell activity and the animal’s upcoming choice can be investigated in a number of ways. First, on a trial by trial (fine-timescale) basis, how well can individual decisions be predicted by splitter activity? Second, across many trials or sessions (long-timescale basis), how does the time course of the splitter signal relate to behavioral performance? In general, relationships between specific firing patterns in the brain and behavior are hard to establish definitively because (1) it’s hard to manipulate only that pattern instead of the entire brain structure, (2) it’s hard to causally ‘write’ specific patterns and observe results (but see de Lavilléon et al., 2015; Robinson et al., 2020). Splitter cells aren’t a physiologically, genetically or anatomically defined population, so are especially hard to target — which means that for now we have been limited to correlational approaches.

First, at the fine-timescale, the basic test is to determine whether the choice made by the animal on a given individual trial (typically left or right on a T-maze) can be predicted by splitter activity on that same trial. Because the very existence of the splitter signal already implies an overall relationship between that activity and behavior, the most informative are error trials. We consider three possible sources of error: (1) the splitter signal becomes inconsistent with experience, for instance if the animal came from the left arm, but the retrospective splitter signal incorrectly registers ‘right’; (2) the splitter signal is degraded or absent (e.g. multiple splitter cells fire in a manner that is inconsistent with each other, or shut down), and (3) the splitter signal is present and correct, but is disconnected from the decision.

Pastalkova et al., 2008 found instances of situation (1): splitter activity during the delay of a continuous alternation task (in a running wheel) sometimes reflected the opposite trial type from what would have been correct alternation. When this occurred, the animal tended to make an error, taking the (incorrect) arm predicted by the delay splitters. Other studies did not try to predict the animal’s specific upcoming choice, but found situation (2): the splitter signal was degraded (less splitting) or the place cell population (including splitter cells) shut down on error trials (Allen et al., 2012; Bahar and Shapiro, 2012; Ferbinteanu and Shapiro, 2003; Robitsek et al., 2013). Thus, these studies generally support a connection between the quality and/or specific content of the splitter signal and behavioral choice.

However, there are also studies that report dissociations between splitter activity and behavioral choice. Catanese et al., 2012 used a continuous T-maze where the animals either alternated left and right choices, or had to choose a cued arm when a cue was present. Crucially, the animal could not predict which trials were going to be cued, and the timing of the cue could be varied. This setup revealed a dissociation between splitter activity and behavioral choice: when the cue was presented ‘late’ on the central stem, but still early enough to have the animals choose the cued arm correctly, splitter activity continued to indicate the incorrect arm until after the decision had been made. A similar approach was used in Ainge et al., 2012 who varied the timing and presence of a discriminative light cue indicating which arm was rewarded. In that case, the average splitter cell activity discriminated the future choice regardless of the characteristics (present/absent, delayed or not) of the cue (only correct trials were analyzed). A possible interpretation of these findings is that the cue-guided response does not depend on the hippocampus, and thus the hippocampal splitter signal, even though it was present and correct, was ignored (situation 3 above).

Second, at the longer timescale of sessions, if splitters were responsible for accurate performance in a task, some of their characteristics should correlate with indicators of performance. As mentioned in the previous section, a number of studies have found that the splitter cell signal develops with training, which generally correlates with behavioral performance. As a result, it can be difficult to untangle whether the increase in splitting is due to experience on the task, and/or due to a more direct role in determining the animal’s choice. A few studies report that once behavior reaches asymptote, the splitter signal continues to increase with additional experience in the same task. For instance, Levy et al., 2021, using calcium imaging in mice, found an increase in the proportion of reliable splitter cells with recording days, while there was no significant difference in performance during that period. A similar effect was seen on the third task in Bower et al., 2005. However, such a partial dissociation between splitter strength and behavioral performance is not necessarily evidence against a causal role for splitters; it may simply be that once the splitter signal reaches a certain strength, it is sufficient to support behavior. In a different study using calcium imaging in mice, the strength of the splitter signal was found to correlate with performance across subjects (Kinsky et al., 2020), but it also increased with experience and this effect was not directly controlled for. Overall, while the amount of experience seems at least an equally good predictor of the strength of the splitter signal as performance in a task, more experiments and analyses are needed to disentangle the influence of these two factors.

Taken together, there is substantial evidence for a relationship between splitter activity and spatial decisions at fine timescales (on a trial-to-trial basis, the quality of the splitter signal decreases in error trials) and moderate evidence at longer timescales (splitter strength increases with performance in some studies, but this could be an effect of experience). The observation that activity during the delay (as opposed to central stem activity) most strongly correlates with subsequent decisions (Pastalkova et al., 2008) is consistent with our interpretation of the Ainge et al., 2007a study in which the hippocampus-dependent delay version shows no splitters on the central stem: the hippocampal-dependent part is to bridge the delay, after which the behavioral policy is outsourced elsewhere, and can be modified (e.g. by a cue) without a corresponding immediate change in the splitter signal if it still exists.

## Computational models of splitter cells and their function

### Models overview

The key feature of splitter cells is that current sensory input (such as that provided at any given position) alone is not sufficient to produce their activity, so that sensory input needs to be augmented with additional information. The first major way to do this is to include a temporal buffer or trace of past and/or future information, an approach used by a family of models that includes **temporal context models** (section *Temporal context models*) and the **successor representation** (section *Successor representation*). A different, non-exclusive possibility is that hippocampal activity reflects the inference of **hidden states**, a process that can be informed by current sensory input but is fundamentally concerned with discovering the structure of the environment and using that structure to classify current sensory input (section *Latent state inference*).

Note that these models operate at the computational and algorithmic levels (Marr, 1982), specifying a computational goal that can be thought of as serving a particular function, along with a recipe for the computational operations performed on the relevant in- and outputs. Thus, they are not only **models of what kinds of processes could generate (reproduce) the splitter cell phenomenon**, but also **proposals about the kinds of purposes** the splitter phenomenon might exist to support. The different models we discuss in this section diverge in their predictions of how splitter cells should behave under various experimental conditions, and although we hint at some of these key features here, the section on *Model predictions and experimental data* below is explicitly concerned with comparing these predictions to existing data and outlining some future experiments aimed at testing them.

### Temporal context models

**Figure 2.**
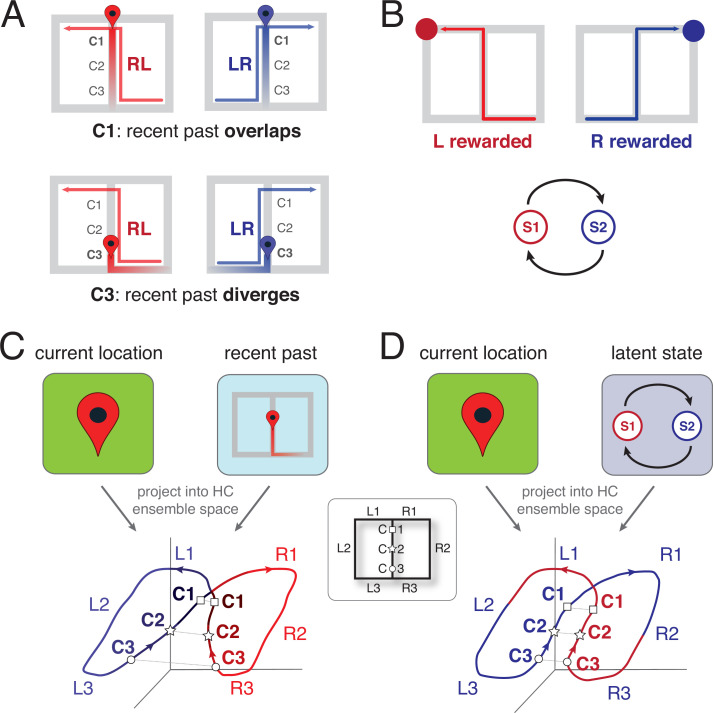
Schematic representation of the two leading theoretical accounts of splitter activity. (**A**) Decaying trace of the recent past during performance of a continuous T-maze alternation (figure-of-eight) task. In this account, the recent past overlaps at location C1 for LR and RL trajectories because of their shared recent past (the central stem; top row). In contrast, at the base of the central stem (C3) the recent past for the RL and LR trajectories diverges. Note that the trace of the past decays gradually, to be stronger for the recent past compared to the distant past. (**B**) Latent task states. In this account, the hippocampus encodes the different states of the task, which for T-maze alternation are two: ‘left rewarded’ and ‘right rewarded’. These states are discrete, and serve to enable the association of state-specific action policies; in this case, the ‘left rewarded’ state specifies that the correct action at the choice point is to turn left. (**C**) Hypothesized neural activity trajectories in principal component space based on the decaying-trace hypothesis in A. Neural activity follows the general figure-of-eight structure of the task itself. Additionally, ensemble similarity between the LR (blue) and RL (red) activity trajectories on the central stem depends on location: at the base of the stem (C3, small circles), activity is more different than at the top (C1, squares) because of the difference in recent past. (**D**) Hypothesized neural activity trajectories for the latent-state hypothesis in B. Unlike the account in C, there is no difference in neural activity similarity along the central stem, because task state switches after information about reward is received (on the side arms). HC: Hippocampus.

The **temporal context model** (TCM) was originally proposed to explain systematic patterns in the order people free-recall items from a sequentially presented list of words (Howard et al., 2005; Howard and Kahana, 2002; Sederberg et al., 2008; for review, see Manning, 2020). At the core of TCM is the idea that experience consists not only of current sensory input, but also includes recently experienced events (or ‘items’) in a short-term memory store (Figure 2c). In TCM, the memory store contains exponentially decaying event traces, but other implementations of short-term memory stores, such as a buffer with a fixed number of slots, will make qualitatively similar predictions. As we will discuss below, having not one, but multiple decay time constants is a further detail that becomes important for certain predictions. Similarly, sequence-learning models of the hippocampus, of which Levy, 1996 is an early example, rely on the idea of a buffer. The events in the memory store get associated together, creating a ‘temporal context’ — a blend of current and past events — that can cue retrieval of items that were previously co-active, and that enables context-sensitive responses to current events (e.g. interpreting the meaning of an ambiguous word depending on the previous sentence).

**Figure 2—figure supplement 1.**
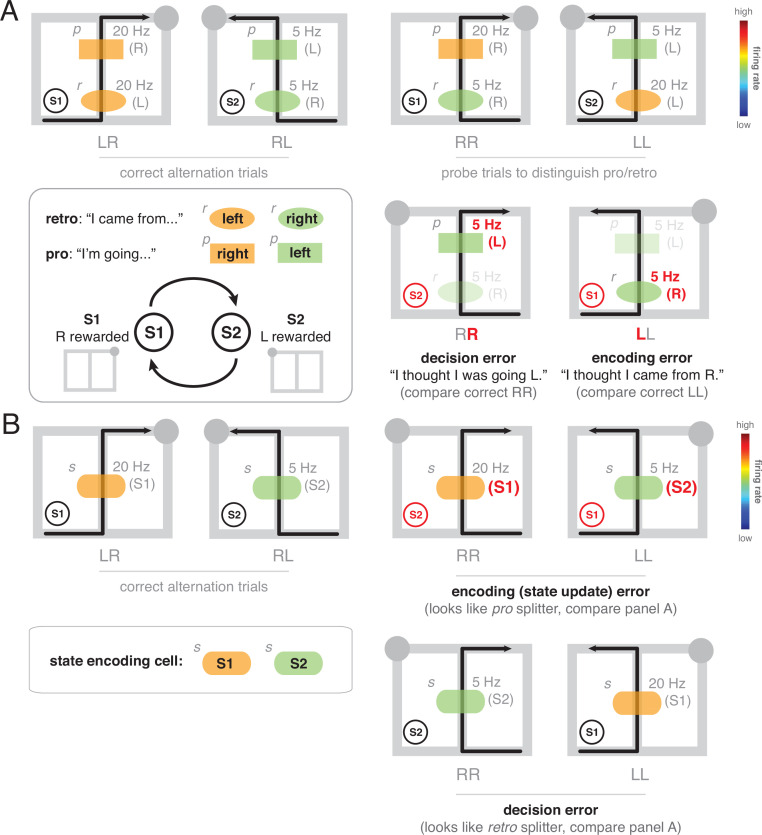
Illustration of sources of error in prospective, retrospective, and state-encoding splitter cells on a continuous alternation task, which has two states, ‘S1’ (right arm rewarded, circle indicates currently rewarded location) and ‘S2’ (left arm rewarded). (**A**) Prospective and retrospective cells do not encode these states directly, but simply track where the subject is going (pro) and where it came from (retro). Thus, a retrospective cell (*r*, oval-shaped firing field) will fire differentially on the central stem for LR and RL trajectories (correct alternation trials, top left; orange indicates high firing rate, green indicates low firing rate) because of the L vs R difference in where the animal came from. Similarly, a prospective cell (*p*, square-shaped firing field) will fire differentially because of a difference in where the animal is going, and so these two types cannot be distinguished on correct alternation trials; note that the *p* and *r* cells have the same activity (colors) for the LR and RL trials. Retrospective cells are revealed by comparing LR and RR (or RL and LL) trajectories (probe trials, top right), and prospective cells are revealed by comparing LR and LL (or RL and RR) trajectories; note that the firing rates (colors) are now different for prospective and retrospective cells. In this scheme, two sources of error are possible. First, the cells may correctly encode what the animal should do, but the decision taken does not match this encoding (**decision error**, note that the prospective cell correctly indicates an upcoming left turn — which is rewarded in S2 - but the animal incorrectly goes R). Second, the cells may incorrectly encode where the animal came from (**encoding error**, note that the retrospective cell fires as if the animal came from R, but actually came from L). (**B**) Splitter cell that encodes S1 (R rewarded) and S2 (L rewarded) directly. Again, two sources of error are possible. First, this cell may incorrectly encode the current state (e.g. fail to update after getting reward), such that when the task is actually in S2, the cell’s activity indicates S1, and vice versa (**encoding error**, top right panels). Alternatively, the cell correctly signals the current task state, but this is ignored in the decision taken (**decision error**, bottom right panels). Surprisingly, for state-encoding cells, encoding errors lead to a pattern of activity that looks like a prospective splitter cell (compare *p* cell in top row of** A**), whereas decision errors lead to a pattern of activity that looks like a retrospective splitter cell (compare *r* cell in top row of **A**). This observation illustrates how the apparently prospective or retrospective character of state-encoding cells is actually an epiphenomenon, resulting from the interaction between task encoding with the task and with the animal’s behavior.

In principle, TCM is agnostic about what the input events to be stored are, and how those events are being encoded (i.e. what ‘features’ of experience they contain). Typical splitter studies involve repeated laps on mazes, which are expected to result in time-varying position, head direction, and high-level feature vectors of sensory input. TCM blends this current experience together with the items in the memory store in a population activity pattern (Figure 2c), such that for a given location, population activity is more similar if the recent past overlaps, and less similar if the recent past diverges (Figure 2a).

TCM offers a natural explanation for retrospective splitter cell activity: on the central stem of a T-maze, past experience is different when coming from the left vs. the right arm (Figure 2a; Hasselmo and Eichenbaum, 2005; Howard et al., 2005). More specifically, a neural ensemble trajectory results that is more different at the base of the central stem compared to the top (Figure 2a, bottom) — a consequence of the temporal context being more different at the base (coming from left vs right for both trial types) than at the top (coming from the bottom for both trial types: more overlap). Thus, TCM explains retrospective splitter cells as a consequence of a decaying memory trace, and predicts a stronger splitter effect closer to the central stem base compared to the top. Interestingly, models of path integration, which share with TCM the basic process of integrating past information to update current state, could be adapted to make similar predictions (e.g. Guazzelli et al., 2001) while other specific models, such as the sequence learning model of Levy, 1996 share similar features and we expect would have similar predictions. To explain prospective splitter cell activity, TCM relies on an associative learning process between the items in memory. A decaying trace of the recent past (e.g. having come from the left) gets associated with the present (turning right), so that after learning, activation of ‘coming from the left’ will contribute to the activation of ‘turning right’.

Note that in this conceptualization, both current location and recent past are *inputs* to the hippocampus, which then combines the two into an ensemble activity pattern realized in splitter cells. This is because in the data, splitter cells do not need to have fields that extend from the side arms into the central arm, or vice versa. Such cells would be the most direct, simplest implementation of a decaying activity trace: after each place cell is activated in its place field, its activity slowly decays to provide temporal context. Splitter cells that lack such a direct trace of past activity must have their memory component realized in some different form, such as an input to the hippocampus (putatively the lateral entorhinal cortex, see e.g. Tsao et al., 2018).

In addition to the encoding stage, a distinct component of TCM is the recall stage, in which temporal context is used to retrieve a memory of what has previously occurred in that context. Applied to the T-maze alternation task, current location on the central stem, supplemented by temporal context of having come from say, the left side, cues retrieval of having turned right and received reward (or any other prior episode). In principle, by the same logic as the retrospective memory trace discussed for the encoding stage, this prospective recall could underlie the activity of prospective splitter cells. We expand on this possibility in the next section covering the successor representation (SR) which is formally related to TCM (Gershman et al., 2012). As above for retrospective memory traces, this retrieved, prospective trace is projected into a hippocampal population activity pattern, such that splitter cells do not need to have a primary field on the left or right arms that extends back into the central stem.

### Successor representation

An idea closely related to TCM is the **successor representation** (SR), which learns to predict future task states from the currently active state (Dayan, 1993; Gershman, 2018). In its simplest form, it answers the question: "given the state I am in now, what state(s) have I previously ended up next?" For instance, given the letter ‘A’, the predicted next letter is ‘B’, because in the past B has tended to follow A. Because the associative learning process in TCM operates on the items co-active in memory, the temporal decay time constant in the (retrospective) memory store in TCM determines how far into the future expected occupancy is tracked. If a trace of past state A is co-active with current state B, A will come to predict B. A will continue to decay as state C appears in memory, causing a weaker association between A and C, and perhaps no association with D at all. Thus, the SR has a ‘temporal horizon’ of how far into the future its predictions extend, with the length of the horizon specified by the temporal discounting parameter. Similarly, multiple possible alternatives are represented probabilistically; for instance, in the central stem of a T-maze, in which the possible next states are ‘left’ and ‘right’, the SR might look like *p(St _t+1_ = left | S_t_ = center)=0.7, p(St _t+1_ = right | S_t_ = center)=0.3* if in the past the animal has chosen the left arm 70% of the time.

Although this idea is straightforward in principle, an important subtlety is critical to its application to splitter cells. As already stated, the SR is learned from past experience, more specifically from the state transitions the animal actually experienced, including those driven by its own decisions (which in reinforcement learning terms is referred to as ‘policy-dependence’). However, the learned SR also critically depends on how states are represented. Consider a T-maze alternation task: when performed perfectly, the expected future occupancies *p(St _t+1_ = left | S_t_ = center*) and *p(St _t+1_ = right | S_t_ = center*) are both 0.5. These probabilities will be the same regardless of when the animal is about to turn left or right, and thus, no prospective splitting would occur. However, if the state representation is expanded to include, for instance, the recent past, then the expected future occupancies diverge: for alternation, *p(St _t+1_ = left | S_t_ = center, S_t-1_=right*) is 1, but *p(St _t+1_ = right | S_t_ = center, S_t-1_=right*) is 0. Thus, the SR cannot, by itself, split the current state based on different expected futures (i.e. result in prospective splitter cells) unless the current state is itself disambiguated (augmented) with past experience or some other discriminating signal.

Regardless of the state representation used to compute the SR, a key characteristic is that expected future occupancy is to be learned from state transitions the animal accumulates over time. Without additional machinery, this ‘running total’ of past state transitions does not adapt quickly to changes in contingencies — a potential source of contrasting predictions compared to other models, as we discuss below. Gradual learning of prospective predictions also contrasts with the retrospective component of TCM, in which traces of the recent past are stored in a decaying temporal buffer which should be available even during the first experience in a given environment, because it does not require learning. The SR has been found to be able to account for a number of experimental findings in the hippocampus (de Cothi and Barry, 2020; Stachenfeld et al., 2017; but see Duvelle et al., 2021) and has recently been applied to splitter cells in a hybrid model that also includes latent state learning to switch between different SRs (Madarasz, 2020).

### Latent state inference

TCM and the SR rely on literal memory of past experience. A different class of model instead uses **latent (hidden) state inference** to decompose the continuously changing (non-stationary) stream of inputs, including not only sensory experience but also the actions taken and the outcomes that result, into states that themselves are associated with a stable (stationary) distribution of those inputs (George et al., 2021; Gershman and Niv, 2010; Niv, 2019; Redish et al., 2007; Sanders et al., 2020; Whittington et al., 2022; Whittington et al., 2020; Wilson et al., 2014; see Fuhs and Touretzky, 2007 for a thoughtful and didactic introduction to latent state models as applied to the hippocampus). Latent state inference echoes the idea of remapping, pervasive in the hippocampal literature (Bostock et al., 1991; Leutgeb et al., 2005; Samsonovich and McNaughton, 1997; Sanders et al., 2020; for reviews see Colgin et al., 2008; Knierim, 2003; Kubie et al., 2020) and has been advanced as a possible explanation for splitters (‘multiple maps’, Kelemen and Fenton, 2016; Lisman et al., 2017; Madarász and Behrens, 2019) as well as many other phenomena. Thus, latent state inference organizes experience into multiple, discrete latent states (or ‘contexts’; Kubie et al., 2020) that do not necessarily correspond to observable states, but instead reflect the agent’s beliefs about the underlying structure of the environment. By contrast, while TCM could be considered a state-based model because it includes information beyond the current sensory input (i.e. a trace of the past, and prediction of the future based on previously experienced transitions), that information was directly observable in the past, and TCM has no inference mechanism for discovering latent (unobservable) state. Thus, we do not consider TCM a *latent* state model. The state inference process includes both the *learning* of appropriate states (the agent’s model of the world), which is a slow process based on extensive experience, and the *selection* of the current state, a fast process based on current evidence.

For instance, in T-maze alternation, the task is designed to consist of two states, arbitrarily labeled S1 and S2, where reward is available on the left in one state (say S1), and on the right in the other (say S2). The task transitions from S1 to S2 only if reward is collected on the left (and from S2 to S1 only if reward is collected on the right). S1 and S2 cannot be observed directly, but must be inferred based on the environment’s response (reward or not) to a given action. If this task structure is recovered, then it could be used to augment information about current location to create the splitter cell effect (Figure 2b, top). Notably, because this hidden state representation does not have temporal decay, this account predicts that the strength of the splitter cell effect is the same along the central stem (Figure 2b, bottom).

The general idea underlying latent state inference is that although raw sensory experience is high-dimensional and dynamic, there is typically an underlying structure to that experience that, if discovered, enables effective prediction of upcoming observations. Of particular importance for a good state representation is that it should enable the agent to predict the consequences of its actions, such as whether a given choice will be rewarded or not. For instance, on a T-maze alternation task, discovery of the two states that make up the task structure enables correct prediction of what follows, say, a left choice: rewarded if the task is in the ‘left rewarded’ state, not rewarded if it is in the ‘right rewarded’ state. The agent needs to discover from experience not only what the states are, but also the transitions between them, i.e. receiving reward on the right transitions the task from ‘right rewarded’ to ‘left rewarded’. Once learned, latent state representations can generalize across tasks where the specific observations may differ, but the underlying structure is the same (e.g. the same alternation task in two different mazes).

A variety of machine learning approaches can learn such task structure from experience, including Dirichlet processes (‘Chinese Restaurant Processes (CRPs)’ which dynamically decide when to split states as more evidence comes in; Gershman and Blei, 2012), Hidden Markov Models (HMMs), and various flavors of deep/recurrent neural networks. For instance, George et al., 2021 use an ‘aliased’ HMM to model splitter cells, and although Whittington et al., 2020 do not simulate splitter cells directly, the ability of their model to predict outcomes based on latent structure suggests their neural network would learn splitter representations as well – recently shown in a preliminary report (Whittington et al., 2022). These models have in common that they learn hidden state representations only to the extent that the learned representations are useful in predicting the future, including outcomes conditional on the agent’s actions. In T-maze alternation, it is crucial to know the state of the task in order to predict whether ‘left’ will be rewarded, whereas knowing the current state is not useful in a free choice task where both are rewarded.

In mapping latent state inference models onto animals solving real-world tasks, an important consideration is that in their most basic form, models like HMMs and CRPs are fit to the *full set* of available observations (i.e. all of the animal’s experience). However, this is unrealistic in practice for several reasons: memory capacity is limited, and models need to be updated dynamically in the light of new observations. To address these limitations, ‘on-line’ versions of such models have been developed, that with each incoming new observation, figure out whether to assign it to an existing state, or to create a new one (Courville et al., 2006; Fuhs and Touretzky, 2007; Gershman and Niv, 2012; Redish et al., 2007). This has the important consequence that the *temporal order* in which experiences are presented influence what is learned. In general, gradual changes promote ‘state-lumping’ (existing states can be updated to accommodate the new, similar observation) whereas abrupt changes promote ‘state-splitting’ (a new, very different observation cannot fit into the existing states, so a new one is created). Experimental support for this idea comes from behavioral experiments that manipulate the abruptness/gradualness of extinction in fear conditioning (e.g. Gershman et al., 2013) and from the order in which ‘morph box’ training proceeds (Leutgeb et al., 2007; Wills et al., 2005). Similarly, if an open environment is experienced from many different angles and trajectories, those experiences will tend to be grouped together into a single state, whereas if experience is forced to be into two very specific, different trajectories, say left-right and right-left on a linear track, those experiences are likely to be split.

The state inference approach uses past experience to learn models that are effective at predicting the future, but during run-time, there is no explicit sense of time: the output of the model is simply what it believes the current state is. Thus, it is not immediately clear how prospective and retrospective splitting can arise from such a state estimate. As a first example, consider the plus maze in Figure 1. The task has two distinct states, ‘W rewarded’ (S1) and ‘E rewarded’ (S2). If S1 and S2 are encoded differently in hippocampal ensemble activity, this would result in ‘prospective’ splitters when starting on the N and S arms, because the animal will tend to choose a different arm depending on current task state (W when in S1, E when in S2). The prospective effect is a consequence of encoding an aspect of the task which is informative for choosing the correct behavior.

A more subtle, but important, example is continuous alternation on a T-maze, where the two task states similarly are ‘left rewarded’ and ‘right rewarded’. As noted earlier, to identify prospective and retrospective cells, error trials are needed; as we show in Figure 2—figure supplement 1, state-encoding cells can have apparent prospective or retrospective correlates, depending on the underlying cause of the error. Specifically, incorrect encoding of task state leads to prospective cells, and disconnects between the (correct) state representation and behavioral choice leads to retrospective cells. The above example highlights how, even though latent state inference is a fundamentally atemporal process (no knowledge of past or future), it can result in activity that relates to upcoming choice, and therefore can appear prospective, as a consequence of interactions with task structure and behavior.

A final consideration to be aware of with state inference models is that the true state space used to set up the experiment may not be the one that the animal learns, and in general there are many possible features that could be included in the learned state representation. For instance, on the plus maze in Figure 1, *task* state, by itself, does not explain retrospective splitters, because say, the W arm is rewarded regardless of whether the animal starts from the N or S arm. However, if the notion of ‘state’ represented by the animal is not limited to *task* state per se, but also includes what the animal needs to know in order to predict how the task will respond to its actions, then the identity of the start arm is relevant, because it determines whether a given action (turn left) will be rewarded or not. This point illustrates the many degrees of freedom that the ‘state’ idea allows, which is a strength but also a challenge when attempting to relate it to experimental data.

## Model predictions and experimental data

The key characteristics of the TCM and latent state models reviewed above suggest a number of domains for model predictions and their comparison with experimental data (summarized in Table 1):

**Table 1.** Summary of model predictions and open questions.

	Temporal context model	Hidden state inference	Data
**Temporal gradedness**	**Y**	**N**	**Y**
**Dissociations between prospective and retrospective splitting**	**Y**: result from different processes (buffer vs. learned from experience)	**Depends**: both occur as a result of the same process, but interacts with task and behavior	Some indications for **Y**, but not tested much
**Increase in retrospective splitting with time**	**N**: no reason why buffer isn’t operational from trial 1	**Y**: state representations need to be learned from experience	**Y**
**More retrospective than prospective splitters even if task fully learned**	?	**Depends:** on task and type of errors	**Y**
**Dependence on task-relevance**	**N**: at least not for retrospective splitters: temporal context is always there	**Y**: bias for representing states that have predictive value	**Mixed evidence**, needs better tests
**Dependence on training procedure**	**N**	**Y**	Generally **Y**, but rarely tested systematically
**Discrete state transitions**	**N**	**Y**	**Untested**
**Splitters generalize across remapped tasks with identical structure**	**N**	**Y**	**Mixed: N** in Bahar and Shapiro, 2012 & Hallock and Griffin, 2013, **Y** in Takahashi, 2013 & Sun et al., 2020

### Temporal gradedness vs. discrete states

The most straightforward feature of TCM is that it employs temporally graded (decaying) representations of the past. This property predicts a non-uniform distribution of splitter fields along the common track, whereby retrospective activity is observed more strongly towards the start of the track (where the recent past splits) and prospective activity more strongly closer to the choice point (where the close future splits; see Figure 2 for a visualization of this idea). Typical splitter cell studies do not report the distribution of fields along the central stem, but a few specific studies have examined this issue (Catanese et al., 2014; Frank et al., 2000; Ito et al., 2015; Ji and Wilson, 2008) and do find the predicted gradients.

In contrast to the *continuous* nature of TCM, latent state models use *discrete* state representations that are switched between. On continuous alternation tasks, the task state switches at the reward sites, and so state-based accounts predict a constant neural trajectory distance, and constant percentages of prospective vs retrospective splitters, along the central stem (Figure 2). Since such temporal gradients are observed in the data, this aspect is not explained by a state-switching account. One possible way in which gradients could result from discrete state switches is if the observed gradient reflects a *distribution* of state switches that occur with a changing probability; however, this explanation seems unlikely for splitter gradients along the central stem of overlapping trajectories, because the task state actually switches at the reward sites (left and right arms), not the central stem. This issue would certainly benefit from more thorough investigation with theoretical models and/or simulations. Thus, the clear evidence for temporal gradedness of the splitter signal across multiple studies is one of the major experimental supports for the TCM theory, while presenting a challenge for accounts that rely on discrete state switching. The temporally graded aspect of TCM also neatly explains why splitter fields are displaced from the central stem to the delay period in delayed alternation, compared to continuous alternation (Ainge et al., 2007a; Griffin et al., 2012; Hallock and Griffin, 2013; Ito et al., 2015; Kitanishi et al., 2021; Pastalkova et al., 2008): the delay should reduce the number of retrospective splitters on the central stem because the recent past becomes more similar following a delay. Similarly, Pastalkova et al., 2008 and Gill et al., 2011 reported that retrospective splitter activity tended to occur at the beginning of a delay rather than towards the end. Experiments aiming at investigating this phenomenon further could use central tracks of increased lengths, for which TCM predicts a stronger divergence of retrospective (at the start) and prospective (at the end) splitter activity while splitter cells resulting from a latent state encoding should be unaffected.

**Figure 3.**
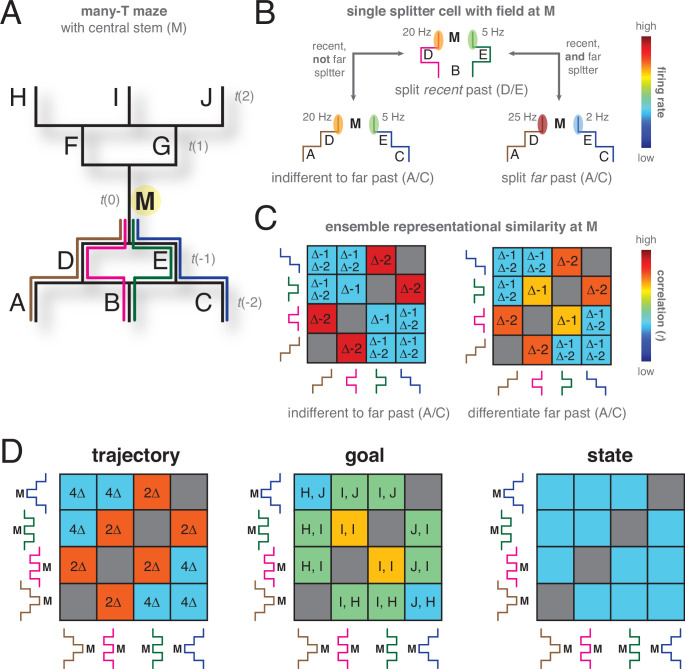
Hypothetical task to illustrate the possible role of representational similarity analyses (RSA) in distinguishing different hypotheses about the content of the splitter signal. (**A**) Task design. A maze (left) that has a single central stem ( M) which can be reached from three possible starting points (segments A-C) through four possible trajectories (brown, magenta, green, blue). Similarly, there are three possible goal locations (segments H-J) with four possible trajectories. Analyses focus on the M segment (*t*=0) so that points A-E are in the past, and F-J in the future. (**B**) On this task, a hypothetical splitter cell at M could distinguish the recent past (D vs E, *t* = –1) while being independent of the far past (same activity as for D vs E, even though A vs C at *t* = –2 are now also different, bottom left panel). Alternatively, its activity could depend on both the recent and the far past (different activity compared to D vs E, bottom right panel). (**C**) Different timescales of encoding traces of the past result in different representational similarity matrices comparing ensemble neural activity at M across different trajectories. Δ–1 indicates comparisons with a difference at t-1, Δ–2 indicates comparisons with a difference at t-2. If only the recent past is encoded in ensemble hippocampal activity (left matrix), trajectories that share the recent segment (no difference at *t* = –1, tiles with no Δ–1 in the matrix) are correlated (red matrix elements), while trajectories that differentiate the recent segment (tiles with Δ–1) are uncorrelated. In contrast, an ensemble that differentiates both recent and far past in a graded manner (right matrix) will show some positive correlation when the far past is shared (no Δ–2, yellow matrix elements). (**D**) Similarly, different possible hypotheses about what is encoded by the splitter cell signal (full trajectory, goal, task state) result in different RSA matrices. For the full trajectory RSA matrix (left panel), correlations are driven by the number of overlapping trajectory segments (high correlation if only 2 segments are different, 2Δ; low correlation if all four segments of the trajectory are different, 4Δ). If ensemble similarity is based on goal (center panel), correlations are driven only by how close in space the goal is (highest for the same goal, I, I; intermediate for adjacent goals such as H, I and J, I; low for far goals). Finally, if neural activity encodes task state (i.e. what trajectory needs to be executed in order to get reward; right panel), every trajectory is equally uncorrelated with every other trajectory, because no spatial information is required to distinguish states. Underlying these RSA predictions is the idea that computations based on state have different information content compared to those based on trajectory: state-based computations, minimally, only require the states to be different. In contrast, trajectories are spatial, and have similarity structure based on factors like distance and amount of overlap.

TCM actually proposes the simultaneous, parallel representation of multiple timescales, that is not just the recent past, but also the more distant past (formally, a range of temporal decay parameters). Prominent theories and data from multiple modalities including tetrode recordings (Tsao et al., 2018) and whole-brain imaging (Baldassano et al., 2017; Honey et al., 2012) support the notion that multiple timescales are represented throughout the brain, but an open issue is if/how this maps onto the hippocampus. Splitter cells offer a unique window into this issue, but to our knowledge their activity has not been analyzed for the existence of multiple timescales. Experiments like Grieves et al., 2016 and Ainge et al., 2007b that use a multiple-T maze such that there are proximal as well as distal differences in past and/or future experience could be used to determine how multiple timescales are represented (see Figure 3 for a schematic); a tantalizing possibility is that this temporal axis maps anatomically onto the dorsal-ventral (posterior-anterior in humans) axis (Keinath et al., 2014; Kjelstrup et al., 2008).

It is important to note that discrete state-switching and continuous temporal gradedness are not mutually exclusive, and can be combined into hierarchically structured representation schemes, where neural activity is grouped into discrete regions of activity space (corresponding to states, maps, or contexts) but can move around continuously within each region (corresponding to temporally decaying representations of experience). In dissecting the relative contributions of these two processes to splitter activity, a potentially useful approach is representational similarity analysis (RSA) which could reveal different distances in ensemble space for states (which have no representational similarity) as opposed to spatial quantities such as trajectories (which do; Figure 3). Similarly, methods that can detect abrupt ensemble transitions in neural activity in time (e.g. Hidden Markov Models, Jezek et al., 2011; Karlsson et al., 2012) could be applied to splitter cell activity to detect if and when such transitions occur.

### Task-relevance, experience clustering, and learning

A crucial feature of TCM is that it is agnostic about task relevance: it represents a temporally graded buffer of the past (and expectations of the future; see the next section) regardless of whether that past is important for solving the task, or not. Note that TCM accepts input features that could include task state (e.g. Polyn et al., 2009) but it has no way of discovering states on its own. Moreover, representations of the past need not be learned; whatever is encoded about current attended experience is available to be stored in the memory buffer. The experimental literature is mixed on this point: on the one hand, in support of TCM, a number of studies have found that retrospective splitters are present even in tasks where the past is not relevant to solving the task. This includes the cued tasks in Ferbinteanu et al., 2011 and Takahashi, 2013 (reviewed in section *Variability of the spliter signal across tasks and studies*) and the reference memory task in Xu et al., 2019. Note that in these studies, the subjects generally performed multiple, different tasks; a rare exception being the retrospective coding observed using calcium imaging in mice in Keinath et al., 2020, in which the activity of place cells differed depending on the entryway into the environment, without any prior experience of an entryway-relevant task.

On the other hand, there are also reports of the **absence** of splitter cells, inconsistent with a major prediction of TCM. Although such negative results are somewhat rare in the literature, perhaps due to publication bias against negative results, they are not unheard of: Lenck-Santini et al., 2001 found no significant splitters during continuous alternation in a Y-maze (but out of only 18 recorded cells); Berke et al., 2009 found no splitter cells on a cued plus maze task; Bower et al., 2005 found no splitters in a trajectory-learning task in an open field; and Griffin et al., 2012 found low percentages (13%) of retrospective splitters during the intertrial interval of a conditional discrimination task. Notably, subjects were only trained on *one task* in all these ‘no splitter’ cases, and these studies shared some other features that we return to below. See also is Gupta et al., 2012 for an example of a lack of splitting in medial entorhinal cortex.

In contrast to the ‘memory buffer’ idea inherent to TCM, latent state inference is more flexible: in the most typical version, only those states/features are learned that are relevant to the task. Thus, if the recent past is irrelevant, as it is in cued tasks where all information required for making a choice is immediately available, the past needs not be represented. This idea predicts differences in splitter signal depending on whether the variable of interest is relevant or not (e.g. random choice vs alternation). As mentioned above, this prediction is generally not a good description of what happens when the same animals are trained on multiple tasks, comparing past-relevant vs past-irrelevant (Ferbinteanu et al., 2011; Takahashi, 2013) because retrospective splitters persist even on cued tasks, but it does account for the apparent reduction or even absence of splitting when animals are only trained on one version of the task where the past is irrelevant (Berke et al., 2009; Griffin et al., 2012; Stevenson, 2011 - PhD thesis).

What are we to make of these apparently mixed results? In particular, why do some tasks where the past is irrelevant nevertheless find splitter cells, but others do not? We believe the properties of latent state inference models could account for some of these differences. As we have already mentioned, some studies that reported little or no splitting only used one task (Berke et al., 2009; Griffin et al., 2012). In this situation, the animals truly have no need for a state space that includes the recent past. In contrast, studies that did report splitting on tasks where the past is irrelevant have generally used multiple tasks that the animals had to switch between (Ferbinteanu et al., 2011; Takahashi, 2013). In this situation, the existence of a memory-dependent task condition has already shown the animal the importance of representing the past, and can be expected to carry over to a task condition where it is irrelevant.

In addition, in latent state inference models, state-splitting is encouraged by highly structured, discrete experiences (e.g. with barriers) and abrupt transitions between them, as occurs when stereotyped trials are batched together in blocks, and then switched to a different set of stereotyped trials. Thus, the abrupt transition from barrier training of a single maze arm to alternation in Lee et al., 2006 would be the canonical case that should create a state split, and indeed that study reports one of the highest percentages of splitter cells in the literature: 67.9% of central stem place cells (see also alternation task in Griffin et al., 2012). Conversely, state-lumping (grouping) is encouraged by the interleaving of continuously varied experiences and gradual changes. Interestingly, interleaving trials makes a reference memory choice task more difficult to learn than when run in blocks (Ainge et al., 2012). Notably, a number of studies reporting the absence of splitters featured continuously changing experience: for instance, on the plus maze in Berke et al., 2009, any goal arm becomes the next start arm, and the next goal arm is pseudorandomized. In contrast, the cued task in Ferbinteanu et al., 2011 was run in blocks of trials where the goal did not change, and discrete trials were used that could only start in the North or South arms. Similarly, Lenck-Santini et al., 2001 used continuous runs in which rats turned around without reward on a Y-maze, and the only version of the Bower et al., 2005 task with no splitters allowed unconstrained trajectories in an open field. Thus, some of the variability across these tasks aligns well with known properties of latent state inference models.

Further support for the latent state view comes from a study where rats had to choose among two objects at the end of the arms of a radial arm maze, with a specific object-location pair being rewarded (the rewarded object depending on the location; Lee and Kim, 2010). In this case, splitter cells seemed to adapt to the task: at first, rats were using (incorrectly) a response strategy to choose the objects and splitter cells encoded the prospective response; with training, performance improved and splitters eventually encoded the object-in-place, regardless of the response used to reach the correct object. Thus, with experience, the rats learned the correct state space for the task, even though there was no change in the actual stimuli being presented.

An important prediction of latent state models is the generalization (re-use) of splitter cell activity across tasks where sensory experience may differ, but the underlying structure is the same: for instance, the same alternation task run on two different T-mazes. The latent states of the two tasks are the same; it is only the mapping between latent states and sensory inputs that needs to change. In contrast, TCM lacks a mechanism for separating sensory inputs from underlying task states, and will by default simply track the sensory observations and their temporal relationships, with no way for generalization. In support of a generalizable state representation, Sun et al., 2020 found that 38% (which was higher than chance) of their ‘lap-counter cells’ generalized from one maze to another (i.e. a cell selective for a specific lap on the square maze also was selective for that lap on a circular maze). Also, Takahashi, 2013 recorded from the same rats in three different tasks, that relied on the same trajectories in the same maze, and found that the trajectory type could be decoded above-chance in a given task from activity in the other tasks. In contrast, Hallock and Griffin, 2013 did not find consistent splitters across two different tasks on slightly different versions of the same maze (even though the same trajectories were involved in the two tasks) and Bahar and Shapiro, 2012 ran three different versions of a plus-maze task (standard place task, place task with goal location changed, place task with local and distal cues changed, while keeping the rules identical) and found that splitting category (prospective, retrospective or even non-splitter) was generally inconsistent between tasks. The mixed evidence for splitter generalization could be because of different strategies being used, that is not necessarily requiring a latent state representation, but perhaps using a place/map-based strategy instead.

### Properties of prospective splitters

TCM maintains not only a temporally graded buffer of the recent past, but also temporally graded expectations of the future. This may be a counterintuitive idea at first, but can be thought of as the result of a simple associative learning rule: “when I’ve been in this location/situation in the past, what happened next?” Because a (decaying) trace of the past is maintained alongside current input, associations form between past and current states, so that activation of the present will cue associative retrieval of upcoming states. More formally, this component of TCM describes a temporally discounted probability distribution over future states given the current state, and is formally similar to the successor representation (SR) in reinforcement learning (Dayan, 1993; Gershman et al., 2012). Prospective splitters are a direct consequence of such a process: if expected future occupancy differs for any experimental conditions (such as the two correct trial types during continuous alternation) then this difference will show up as a splitter signal. Moreover, because the SR/TCM is temporally discounted, it predicts a gradient where the splitter signal on the continuous T-maze is stronger closer to the choice point (because the possible upcoming futures are more different) compared to the base of the central stem (where the possible upcoming futures are more similar since they share more of the same stem). More generally: prospective and retrospective activity should be observed before and after a choice point respectively, and decline in prevalence/strength with distance from that choice point.

However, the existence of prospective splitters itself is not a unique prediction of TCM/SR: encoding of task state, trajectory, and/or policy representation would similarly result in prospective splitters. As mentioned earlier, although latent state models do not represent expectations about the future explicitly, they do represent states that the animal uses to make decisions about the future, which could appear as prospective cells. In addition to the predicted temporal gradient, TCM/SR does have some further features that could be used to distinguish it from such alternatives: the SR should group together situations with a different past but shared future, such as occurs on plus mazes when comparing say, NE and SE trajectories: SR predicts splitter cells with similar activity on the N and S arms because of the shared E future, compared to say, NE and SW (in this case, those trajectories also share the same goal; further controls would be needed to rule that out). However, Guise and Shapiro, 2017 did not find evidence for such common-goal coding. Further unique predictions of the SR account include: (1) it should represent multiple timescales simultaneously, not only the upcoming choice outcome (see Figure 3 for a visual representation of this idea), (2) it should encode expectations of future occupancy that span a larger number of future trajectories, not just a binary left/right comparison (e.g. on double-Y mazes, or radial arm mazes).

### Dissociations between retrospective and prospective splitters

In TCM, retrospective splitters and prospective splitters arise through different processes: retrospective splitters don’t require learning, but rather result from a decaying working memory buffer, and should therefore always be present. In contrast, prospective splitting results from the learning of experienced transitions, yielding the successor representation. Thus, if prospective splitters reflect the SR, then they should develop more slowly with experience compared to retrospective splitters, which are a ‘free’ and immediate consequence of having a trace of the recent past. Furthermore, a splitting SR depends on having relevant features of the past represented — for instance, on continuous alternation, if there is no representation of the previous trial, there is no way for the expected future to depend on that representation. The decaying memory traces of TCM can in principle provide the required information about the past, but whether this is sufficient in practice to learn the SR on continuous alternation tasks would need to be explored with simulations. More generally, the SR and latent state representations can interact, a point that will be relevant to our general conclusions in the next section (*Conclusions and remaining open questions*). In any case, there are a number of predictions from this account that are yet to be tested experimentally.

In hidden state inference models, retrospective and prospective splitting are a side effect of a learned state representation and so typically develop with experience, unlike the more automated retrospective splitting in TCM. Because fundamentally this type of model does not distinguish between prospective and retrospective splitting, it does not have a mechanism for having these types develop at different rates. Instead, whether prospective and/or retrospective splitters are seen would depend on the animal’s structuring of the task. For instance, in the plus maze task of Ferbinteanu and Shapiro, 2003 the two *task states* are E rewarded (S1) and W rewarded (S2). If the animal learns a state representation limited to these task states, this would result in prospective but not retrospective splitters (when starting from the south arm, S1 will be active for SE trajectories, and S2 for SW, leading to prospective splitting; but S1 will be active for NE as well as SE, so no retrospective splitting). Alternatively, a more useful state representation that can uniquely specify the best action would include different learned states for NE vs SE as well (generating retrospective splitting), because the action to be taken is different (turn left vs. right).

Across studies, a clear pattern is that there is stronger retrospective splitting compared to prospective splitting (but note that this was not replicated in a model of splitter cells used to drive a robot; instead, more prospective cells than retrospective cells were predicted, Fleischer et al., 2007). In CA1 (and CA3), every paper surveyed reported a greater population of retrospective than prospective cells (Bahar and Shapiro, 2012; Catanese et al., 2014; Ferbinteanu et al., 2011; Ferbinteanu and Shapiro, 2003; Frank et al., 2001; Frank et al., 2000; Shin et al., 2019; Tang et al., 2021). On average, more than twice as many retrospective cells than prospective cells are observed. In addition to single-cell analyses, both Tang et al., 2021 and Ito et al., 2015 found greater decoding accuracy for retrospective paths than prospective ones in CA1. The greater prevalence of retrospective coding in the hippocampus is observed even on plus mazes, where in state space terms, the origin arm (as encoded in retrospective splitters) is irrelevant by the time the animal is on the destination arm, and such models would favor prospective splitting. Regarding extra-hippocampal areas, in the mEC (Frank et al., 2000; Frank et al., 2001), anterior cingulate cortex (Procyk et al., 2000), OFC (Young and Shapiro, 2011) and mPFC (Shin et al., 2019; Tang et al., 2021), on average 50% more retrospective splitters are observed than prospective ones; specifically in mPFC and NRe, Baeg et al., 2003; Ito et al., 2015; Tang et al., 2021 also found greater decoding accuracy for past trajectories than future ones; however, in a continuous W-maze task where animals returned to the central arm via a shortcut rather than retracing their path through the maze, Fujisawa et al., 2008 found evidence of prospective differential activity in the mPFC, but very little evidence for retrospective activity.

The exact explanation for this difference is unclear at this point, but we offer a few speculative possibilities. The fact that the SR (putative prospective component), but not the retrospective component, needs to be learned from experience is one possible source; however, in many studies, the animals have extensive experience with the task, and therefore this explanation seems unlikely (but would fit with a SR-like representation being a consequence of the over-learning of routes). However, there is a fundamental asymmetry between the past and the future: the past is known completely, whereas the future is uncertain to varying degrees. Depending on the encoding scheme used (Ujfalussy and Orbán, 2021), this may explain the stronger retrospective splitter signal in TCM. Alternatively, cells that encode task state can interact with behavior to produce apparent retrospective coding, specifically when a correct state signal is overridden by an alternative strategy (see Figure 2—figure supplement 1 for a worked out explanation), as occurs in, for instance, Catanese et al., 2014.

## Conclusions and remaining open questions

### Summary

The study of splitter cells provides one of the clearest demonstrations of internally generated cognitive processes fundamental to hippocampal operation because they examine neural coding on-line, during task performance, yet control for sensory input. The first contribution of this review is that we have synthesized findings from two decades of experimental studies that have used a variety of behavioral tasks and training procedures, different analysis methods, and different terminology. We identified patterns that held across different studies, and examined possible reasons for differences across them. In this effort, we built on previous reviews that have covered similar ground but are due for updating (Ainge et al., 2008; Dudchenko and Wood, 2014) or others that show a more tangential interest for splitter cells in the context of a different question (Alexander et al., 2020; Banquet et al., 2021; Griffin, 2021; Nyberg et al., 2022; Ranganath, 2019; Shapiro et al., 2006).

Our second contribution is that we translate between single-cell and ensemble perspectives. Applying the ensemble perspective to studies which have not explicitly done so establishes a common framework for unifying diverse past experiments, for comparing different hypotheses (e.g. as we do in Figures 2 and 3), for comparing across different types of data (spikes, calcium imaging, human neuroimaging) and for comparison with theoretical and computational ideas about hippocampal function. While individual experimental studies (most notably Takahashi, 2013; see also Keinath et al., 2020), have taken this view, these have been the exception rather than the rule, and no reviews have systematically applied it.

Our third contribution, and the most novel one, is that we examined the combined findings from these studies through the lens of two non-exclusive theoretical ideas: temporally graded traces of past and/or future (temporal context), and latent/hidden state inference. These theories are two of the leading computationally motivated proposals that link hippocampal function with specific cognitive processes, and the focus of a number of highly influential papers (Howard and Kahana, 2002; Niv, 2019; Stachenfeld et al., 2017; Whittington et al., 2020). Although these studies made detailed predictions about various forms of hippocampal activity, which have been compared with, and in many cases motivated, specific experimental studies, a direct comparison of their specific predictions in the well-constrained domain of splitter cells has to our knowledge not been performed; yet, as we hope we have shown, splitter cells are in fact an ideal, highly informative domain for testing and refining these theories.

In particular, we find that the unique signature properties of each of the temporal context and latent state inference models are necessary to account for features of data, but that neither theory, by itself, can account for all the data. In fact, several well-replicated experimental findings contain clear patterns that present major challenges for each theory. Specifically, the **temporal gradedness** of the splitter signal on overlapping segments (more retrospective early, more prospective late; shifted backward when delays are added) is strong support for TCM, and difficult to explain using switches between discrete state representations. On the other hand, the **flexibility** of the splitter signal, as indicated by its apparent dependence on training history and task-relevance, is naturally explained by state inference models, while presenting a challenge for TCM. Crucially, these two model classes are not mutually exclusive, and in fact might interact in productive ways that are now beginning to be explored in “hybrid” models (Geerts et al., 2022; Gershman et al., 2014; Madarász and Behrens, 2019; Whittington et al., 2020).

In addition to this overall picture, the viewpoint offered by these theories clarifies the possible interpretations and functions of prospective and retrospective splitting. In TCM, these are truly temporally forward- and backward-looking, but are expected to emerge at different rates because the prospective component (successor representation) has to be learned from experienced transitions, whereas all that is required for the retrospective component is a memory buffer, presumably available at any time including the first trial of a new task. Latent state theories, on the other hand, have no explicit notion of time, but form states in accordance with their value in explaining task observations and reward. A prospective or retrospective appearance is an epiphenomenon induced by the structure of the task and by whether or not the splitter signal is used to guide behavioral decisions (Figure 2—figure supplement 1 see also section *Dissociations between retrospective and prospective splitters*). In any case, these two theories point to a number of domains for new analyses and experimental designs, as we discuss in the next section.

### Open questions and next steps

We see four main areas for open issues that seem timely and productive for future work to address:

- Experimental and computational characterization of differences between prospective and retrospective splitting. TCM and latent state theories make different predictions about the speed at which these emerge with learning. TCM predicts retrospective first, prospective later, whereas state-splitting accounts do not differentiate them and should emerge simultaneously. Experiments should study the time course of both types in experiments that can dissociate them without relying on error trials (i.e. not the classical continuous T-maze). On the theory side, computational models that seek to explain hippocampal activity have generally not considered prospective and retrospective splitting separately, even though there are some consistent patterns in the data that have been replicated across experiments, such as temporal gradients and the generally higher proportion of retrospective compared to prospective splitters.
- Temporal horizon and timescales. What is the temporal horizon of the splitter phenomenon, and how are multiple relevant timescales represented? Experimental studies of the splitter phenomenon have generally focused on tasks with one branch point (like T, W, and plus mazes). Those rare tasks that have included more than one branch point Ainge et al., 2007b; Grieves et al., 2016 have not analyzed the data with this question in mind, and no joint comparisons across (sub)regions and timescales have yet been made.
- Comparisons across learning, and across tasks specifically designed to encourage and/or inhibit state splitting. A core feature of latent state accounts is that the states that are learned depend on the specific temporal order and task-relevance of previous experience. In agreement with this, some experimental results suggest that the existence and strength of the splitter signal depends on the animal’s past experience and specific sequence of task training (reviewed in section on *Task-relevance, experience clustering, and learning*, e.g. Bower et al., 2005; Lee et al., 2006) but this idea has not been systematically tested.
- Understanding the factors that drive splitting using representational similarity analysis. Information about trajectories and goals is spatial in nature, and therefore expected to have a representational similarity structure that reflects spatial relationships (e.g. nearby goals or overlapping trajectories are represented more similarly). In contrast, states do not need such information content in order to achieve their most basic function, although they may be arranged hierarchically or in some other structure. This RSA approach is particularly well-suited to cross-species or cross-methods comparisons (e.g. electrophysiology vs fMRI, rat vs humans).

We also propose a number of bigger picture domains for longer-term research programs to address. The first is to understand how the splitter phenomenon is organized anatomically – within subfields of the hippocampus and subregions and layers of the entorhinal cortex – and its dependence on interrelated brain structures such as medial prefrontal cortex and orbitofrontal regions (see Box 1). Second, as we have illustrated repeatedly, state space models are powerful but also very flexible: what they learn depends on the animal’s priors and specific experience, and for any given task, multiple different state spaces could be learned. Understanding individual differences, their roots in past experience, and consequences for what strategies and structures are learned will be important issues moving forward.

To conclude, it is natural to wonder why it should be that, apparently, two distinct and on the face of it, quite different theoretical notions of temporal context and latent state inference are needed to account for experimentally observed properties of the splitter signal in the hippocampus. We speculate that this is because these two processes likely need each other. Specifically, inferring latent states from experience benefits greatly from memory at various timescales, to detect changes compared to recent experience, or to perform a kind of model comparison on accumulated data. Conversely, building effective predictions for the future, as instantiated in the SR, requires correctly classifying the current state. For instance, on spatial alternation, the SR from a non-augmented ‘central stem’ state would be symmetric for left and right; but the SR for ‘central stem having come from the right arm’ would be (correctly) biased to the left (Madarász and Behrens, 2019).

In any case, we hope to have generated renewed interest in the splitter phenomenon as an informative window into the representations and computations that are core to hippocampal function and cognition more generally, while stimulating future work at the interface of experimental and theoretical neuroscience.
